# Third-Order Neurons in the Lateral Horn Enhance Bilateral Contrast of Odor Inputs Through Contralateral Inhibition in *Drosophila*

**DOI:** 10.3389/fphys.2019.00851

**Published:** 2019-07-09

**Authors:** Ahmed A. M. Mohamed, Bill S. Hansson, Silke Sachse

**Affiliations:** Department of Evolutionary Neuroethology, Max Planck Institute for Chemical Ecology, Jena, Germany

**Keywords:** olfaction, calcium imaging, *Drosophila*, odor processing, contralateral inhibition

## Abstract

The survival and reproduction of *Drosophila melanogaster* depends heavily on its ability to determine the location of an odor source and either to move toward or away from it. Despite the very small spatial separation between the two antennae and the redundancy in sensory neuron projection to both sides of the brain, *Drosophila* can resolve the concentration gradient by comparing the signal strength between the two antennae. When an odor stimulates the antennae asymmetrically, ipsilateral projection neurons from the first olfactory center are more strongly excited compared to the contralateral ones. However, it remains elusive how higher-order neurons process such asymmetric or lateralized odor inputs. Here, we monitored and analyzed for the first time the activity patterns of a small cluster of third-order neurons (so-called ventrolateral protocerebrum neurons) to asymmetric olfactory stimulation using two-photon calcium imaging. Our data demonstrate that lateralized odors evoke distinct activation of these neurons in the left and right brain hemisphere as a result of contralateral inhibition. Moreover, using laser transection experiments we show that this contralateral inhibition is mediated by presynaptic neurons most likely located in the lateral horn. Finally, we propose that this inhibitory interaction between higher-order neurons facilitates odor lateralization and plays a crucial role in olfactory navigation behavior of *Drosophila*, a theory that needs to be experimentally addressed in future studies.

## Introduction

Chemotaxis is important for the survival of many animals since chemicals that are emitted by the environment can be exploited as cues for potentially positive (e.g., food, mate, or oviposition site) or negative (toxicity, competitors, predators, or parasitoids) interactions. Especially insects rely heavily on their sense of smell to ensure survival and reproduction and have, in most cases, a highly developed and sophisticated olfactory system. To navigate toward (or away from) an odor source, walking and flying insects usually use multiple strategies. Besides anemotaxis, one of the most used strategies is to detect an odor gradient across the two antennae by comparing their signal strength, and to turn toward or away from the stronger olfactory signal, a phenomenon termed “osmotropotaxis” (Martin, 1965; Hangartner, 1967; Kennedy and Moorhouse, 1969). Confusing this strategy, by spatially reversing the antennae (i.e., by crossing and fixing them), impairs chemotaxis as shown in bees, ants, and locusts (Martin, 1965; Hangartner, 1967; Kennedy and Moorhouse, 1969). The vinegar fly *Drosophila melanogaster* also uses the same strategy to navigate toward or away from an odor source (Borst and Heisenberg, 1982; Duistermars et al., 2009; Gaudry et al., 2013). At the neuronal level, the peripheral olfactory system (i.e., the antennae) of vinegar flies responds differently to lateralized odors (i.e., a bilaterally asymmetric odor stimulation) compared to symmetric odor application (Louis et al., 2008; Duistermars et al., 2009). However, little is known about where the information from both antennae becomes integrated and how higher brain centers process asymmetric odor stimulation to ensure reliable navigation toward odors.

The olfactory circuitry of *Drosophila* has been fairly well characterized, making it a premier model system for studying odor processing strategies. Sixty-two odorant receptors (ORs) are expressed in the dendrites of olfactory receptor neurons (ORNs) (Clyne et al., 1999; Vosshall et al., 1999). The ORNs are housed in hair-like structures called sensilla on the peripheral olfactory appendages (i.e., the antennae and maxillary palps) and express usually one (sometimes two) OR type each. The axons of all ORNs expressing a given OR type converge onto the same glomerulus in the antennal lobe (AL, the analogous to the vertebrate olfactory bulb) (Vosshall et al., 2000; Couto et al., 2005). ORNs synapse onto second-order neurons, so-called projection neurons (PNs, analog to mitral/tufted cells in vertebrates). The axonal terminals of PNs relay the olfactory information from the AL to two higher-order neuropils, which are the mushroom bodies (MBs) (analogs to the piriform cortex in mammals), representing a center of learning and memory, and the lateral horn (LH) (analogous to the mammalian amygdala) that mediates predominantly behaviorally innate responses (Dubnau et al., 2001; Heimbeck et al., 2001; Heisenberg, 2003; Strutz et al., 2014). Third-order neurons, such as MB and LH output neurons (MBONs, LHONs) convey the information to next level protocerebral regions, as, e.g., the ventrolateral protocerebrum (VLP) whose functions remain, so far, largely elusive.

In *Drosophila*, unlike most insects, the majority of ORNs projects from the antennae bilaterally to both brain hemispheres (Stocker et al., 1990; Couto et al., 2005). This bilateral redundancy in morphology may imply that odor inputs are symmetrically directed to both hemispheres. However, the input from the left and right antennae is coded distinctively since ORNs release an asymmetric amount of neurotransmitters in the ipsi- and contralateral AL (Gaudry et al., 2013). As a consequence, the ipsilateral PNs are 30–50% more strongly activated by asymmetric bilateral odor input than the sister neurons in the contralateral AL (Gaudry et al., 2013). In addition, odor lateralization has also been demonstrated at the synaptic level in the AL. Neuronal tracing from serial electron microscopy sections showed that PNs of a given glomerulus share a higher number of synapses with the ipsilateral ORNs than with the contralateral ones (Tobin et al., 2017). Hence, odor input from the ipsi- and contralateral antenna seems to be coded in different ways at the AL level.

In order to study how lateralized odors are processed by higher-order neurons, we investigated the neuronal responses of a specific cluster of LHONs, so-called VLP neurons (hereafter VLPn), to asymmetric and symmetric odor stimulations using two-photon functional imaging. We found that odor-evoked responses of VLPn were suppressed when an odor was presented to the contralateral side. Hence, the detection of an odor gradient is accomplished in a way that asymmetric odor stimulation suppresses the responses in contralateral VLPn. Notably, the observed contralateral suppression is not induced by VLPn in the contralateral hemisphere, but, most likely, mediated by presynaptic neurons located in the LH. Our data demonstrate for the first time that higher-order neurons respond distinctively to a lateralized odor stimulus through contralateral inhibition and therefore enhance the contrast of odor concentration gradient between both brain hemispheres.

## Results and Discussion

### Contralateral Stimuli Suppress Odor Responses in VLP Neurons

**FIGURE 1:**
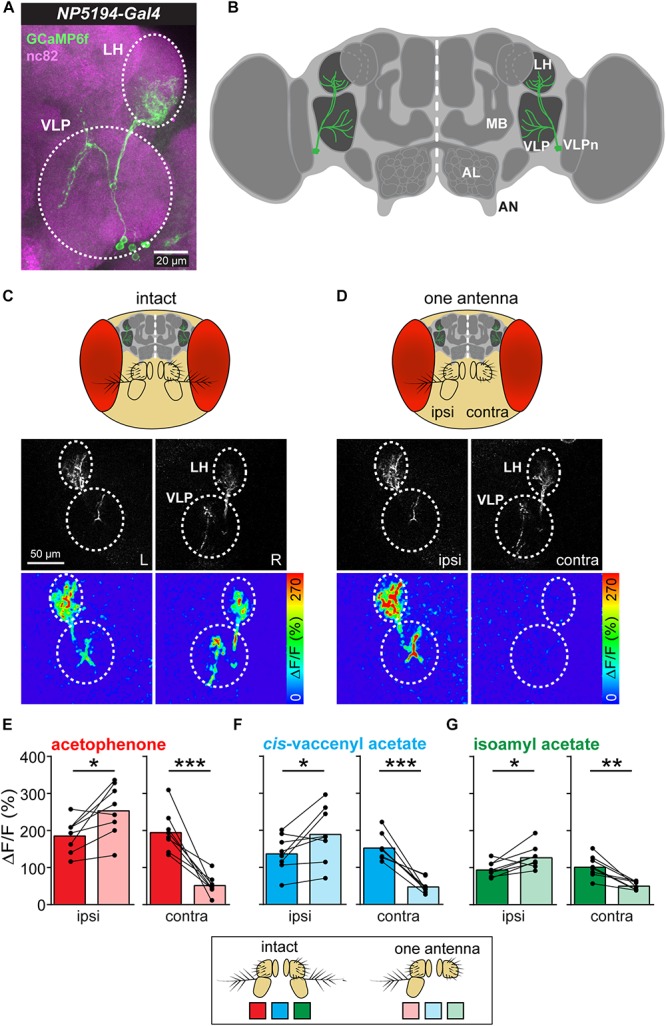
Asymmetric odor stimulation induces contralateral suppression. **(A)** Confocal z-projections of VLPn labeled by *NP5194-Gal4* in an adult female brain. Labeling of GFP (green) and the neuropil marker nc82 (magenta) are shown. Dashed circles represent the lateral horn (LH) and ventrolateral protocerebrum (VLP). Scale bar = 20 μm. **(B)** Schematic illustration of the *Drosophila* brain showing the innervation pattern of the VLPn in the LH and the VLP. Mushroom body (MB), antennal lobe (AL), and antennal nerve (AN) are shown for orientation. **(C,D)** *Upper panel*: Schematic head of *Drosophila* with intact antennae **(C)** and after removal of the third antennal segment of one antenna **(D)**. *Middle panel*: Gray-scale image represents the VLPn structure expressing GCaMP6f. Dashed circles indicate the LH and the VLP in the left (L) and right (R) brain hemispheres. Scale bar = 50 μm. *Lower panel*: false-color coded images showing odor-evoked responses from a representative animal before **(C)** and after **(D)** antennal removal. Dashed circles represent the LH and VLP. **(E–G)** Calcium signals obtained with two-photon imaging from flies bearing *UAS-GCaMP6f* under control of *NP5194-Gal4* from the ipsi- and contralateral sides (to the intact antenna) before and after antennal removal to stimulation with acetophenone **(E)**, *cis*-vaccenyl acetate **(F)**, and isoamyl acetate **(G)**. Dots and lines represent individual flies, bars represent the mean (*n* = 8; ^*^p < 0.05; ^∗∗^*p* < 0.01; ^∗∗∗^*p* < 0.001, paired *t*-test).

Ventrolateral protocerebrum neurons have their postsynaptic dendrites in the LH and send their axonal terminals to the VLP, where they synapse onto further higher-order neurons (Figures 1A,B). Previous studies have shown that these third-order neurons receive input from olfactory PNs in the LH and respond to a variety of different odors (Liang et al., 2013; Strutz et al., 2014). Furthermore, VLPn exhibit a stereotypic innervation pattern in both neuropils and are involved in innate odor-guided behavior (details will be described in Mohamed et al., in preparation). To investigate how these third-order neurons respond to lateralized odors, we measured their responses to symmetric and asymmetric odor stimulations. In order to provide an unilateral odor input, we surgically ablated one antenna and monitored odor-evoked calcium signals at the two-photon microscope of VLPn of the ipsi- as well as contralateral brain hemisphere to the intact antenna before and after antennal removal (Figures 1C,D). To selectively measure VLPn, we expressed GCaMP6f under control of the enhancer-trap line *NP5194-Gal4* (Jefferis et al., 2007), which labels a subpopulation of these third-order neurons (cell count = 4.8125 ± 0.1875 neurons). Surprisingly, we observed an asymmetry of the odor-evoked signals between the ipsi- and contralateral side to the odor before and after antennal removal. On the one hand, the calcium signals to the odor acetophenone were significantly increased in VLPn on the ipsilateral side to the intact antenna after removing the contralateral antenna compared to bilateral stimulation (i.e., with intact antennae) (Figure 1E). On the other hand, the odor-evoked responses were strongly reduced in contralateral VLPn to the intact antenna. Hence, our results suggest that VLPn receive a contralateral inhibition in response to asymmetric stimulation with the odor acetophenone. To test whether this contralateral suppression was odor-independent, we used the food odor isoamyl acetate (Schubert et al., 2014) and the male-specific sex pheromone *cis*-vaccenyl acetate (Kurtovic et al., 2007) as additional olfactory stimuli. As expected, VLPn on the ipsilateral side to the odor showed a similar contralateral inhibition to asymmetric stimulation with these two odors. This inhibition is characterized by a significantly increased ipsilateral response to the odor after removing the contralateral antenna (Figures 1F,G). Since Duistermars et al. (2009) has reported that sensory signals from the left antenna contribute disproportionately more to odor tracking than signals from the right side, we sought to analyze whether the observed contralateral inhibition is different between both brain hemispheres. However, we could not find any significant difference between the odor-evoked calcium responses of the right and left sides (data not shown) indicating that the contralateral inhibition is not side-specific.

As mentioned above, lateralized odors are coded at the PN level in a way that ipsilateral PNs are more strongly activated by an asymmetric odor stimulus than their contralateral sister PNs (Gaudry et al., 2013). This asymmetry in PN responses can be attributed to two main mechanisms: First, the release of neurotransmitter at the ORN-to-PN synapse in the AL is asymmetric (Gaudry et al., 2013), and second, PNs have significantly more synapses with ipsilateral than with contralateral ORNs (Tobin et al., 2017). The last finding is similar to the mechanosensory system of leeches, where individual mechanoreceptor neurons exhibit a higher number of synapses with the ipsilateral postsynaptic neurons than with the contralateral sister cells (Lockery and Kristan, 1990) enabling the animal to detect the stimulus orientation. Also the mammalian olfactory system processes the input from both nostrils separately. In the periphery, odor responses within the olfactory epithelium as well as odor-evoked intrinsic signals at the glomerular layer in the olfactory bulb of rats reveal strong olfactory lateralization (Parthasarathy and Bhalla, 2013).

Our results provide evidence that odor inputs are distinctively encoded in the two hemispheres at the level of third-order olfactory neurons of *Drosophila*. Notably, we observed that an asymmetric odor stimulation does not only activate the ipsilateral VLPn to the intact antenna significantly more strongly than their contralateral sister neurons, but also leads to a contralateral suppression (i.e., the ipsilateral VLPn were more strongly activated by a lateralized odor stimulus than a symmetric stimulus). This finding is reminiscent of the visual system, where higher-order neurons exhibit a contralateral inhibition when stimulated with an asymmetric visual stimulus (Shiozaki and Kazama, 2017; Sun et al., 2017). However, in contrast to our results, visual neurons show a strong inhibition to a contralateral stimulus, while VLPn reveal no, or only a very weak, response to contralateral odor stimulation. This finding can be explained by the fact that the contralateral PNs to the odor input still become strongly activated due to the bilateral projections of their cognate ORNs (Gaudry et al., 2013). This PN activation would in turn result in an excitation of the contralateral VLPn and therefore, due to the contralateral inhibition, compensate the PN input.

Taken together, our results show that olfactory input from both antennae leads to a contralateral inhibition in a subset of olfactory third-order neurons which seems to be odor-independent.

### Contralateral Inhibition Occurs Presynaptic to VLPn

**FIGURE 2:**
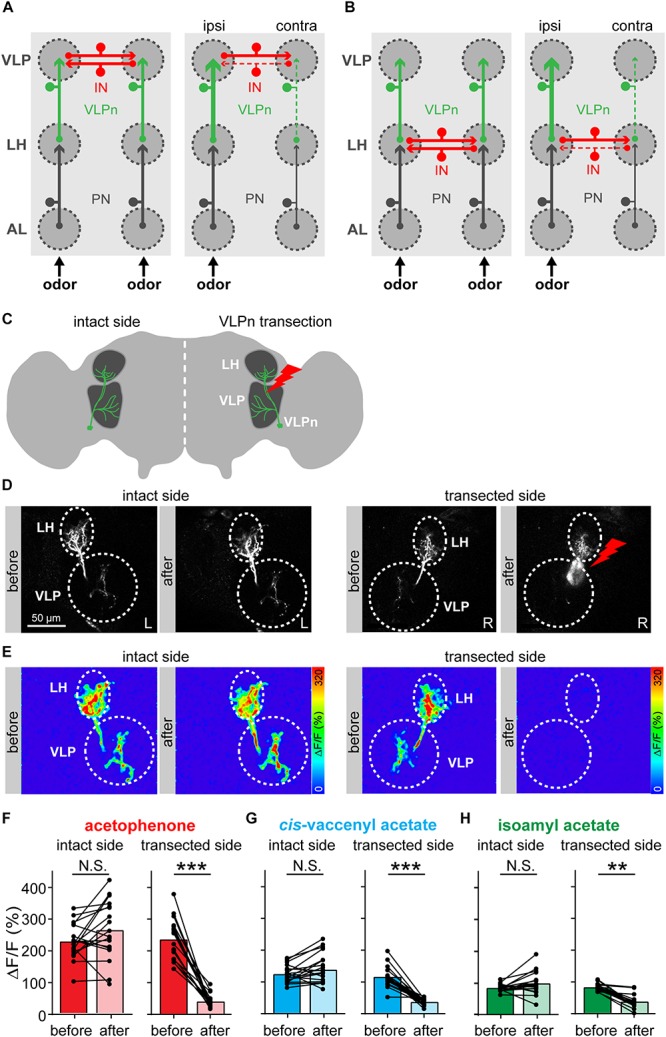
Contralateral inhibition takes place presynaptically to VLPn. **(A,B)** Two proposed models for the contralateral suppression in VLPn. In both models, projection neurons (PNs, black) activate downstream the VLPn (green). Presynaptic input is represented by an arrow, postsynapses are indicated by a small circle. In the VLP inhibition model **(A)**, inhibitory neurons (INs, red) connect the VLPn of both sides at their postsynaptic sites (i.e., within the VLP neuropil). In this case, activation of VLPn on one side would be required to induce inhibition in the contralateral VLPn. In the LH inhibition model **(B)**, the IN (red) connects the VLPn of both sides at their presynaptic sites (i.e., in the LH). Here, activation of VLPn would not be required to cause contralateral inhibition. In both models **(A,B)**, the strength of activation is represented by the line size; solid lines represent active neurons and dashed lines represent not activated and/or inhibited neurons. **(C)** Schematic drawing of the laser transection experiment. VLPn were transected only on one brain side, while leaving VLPn on the contralateral side intact. This allowed us to abolish any odor-evoked responses of VLPn in the transected side. **(D)** Gray-scale images of VLPn expressing GCaMP6f under control of *NP5194-Gal4* before and after laser transection in the intact and transected side. Scale bar = 50 μm. **(E)** Examples of false-color coded images of odor-induced Ca^2+^ signals corresponding to each gray-scale image shown in **D** from a representative fly. **(F–H)** Paired comparisons of the calcium signals before and after the transection of VLPn across different flies. Odor-evoked Ca^2+^ signals were recorded and analyzed for both sides (i.e., intact and transected side). Ca^2+^ signals were abolished in VLPn in the transected side after transection. The intact brain hemisphere shows a slight, but not significant, increase prior to the transection. Dots and lines represent individual flies, bars represent the mean (*n* = 18; ^*^p < 0.05; ^∗∗^*p* < 0.01; ^∗∗∗^*p* < 0.001, paired *t*-test).

We next wondered how this contralateral inhibition in VLPn is induced. We envisioned two different circuit models that could account for it. In the first model, we propose that the contralateral inhibition is taking place at the VLP level and is mediated by inhibitory neurons connecting the ipsi- and contralateral VLP neuropils. This model is supported by the fact that VLPn possess presynaptic densities in the VLP, but hardly in the LH (Mohamed et al., in preparation) (Figure 2A). We termed this model “VLP inhibition.” In the second model, termed “LH inhibition,” we hypothesize that the inhibitory neurons are located in the LH resulting in a contralateral inhibition of the excitatory PN input to VLPn (Figure 2B). Notably, such neurons that connect the LH of both hemispheres have been previously reported (referred to as PV12a1) and express GABA as a neurotransmitter (Dolan et al., 2018). In order to test these two hypotheses, we silenced the VLPn in one brain hemisphere by laser transection, while keeping the other side intact and monitoring the odor-evoked responses before and after VLPn manipulation (Figures 2C,D). If the “VLP inhibition” model were correct, transecting VLPn of one side would increase the activation of the contralateral neurons to the transection, as the transected side would fail to activate the inhibitory neurons and therefore contralateral suppression to odor input would not occur. Alternatively, if the “LH inhibition” model were true, transecting VLPn on one side would not affect the activation of the contralateral VLPn, since the contralateral suppression would have occurred prior to the VLP neuropil. When we compared the VLPn responses to odor stimulation with acetophenone, isoamyl acetate, and *cis*-vaccenyl acetate before and after transection on either side, the calcium responses remained constant on the contralateral side, while the response was almost abolished on the transected side (Figures 2E–H). Since we did not observe any response increase after transection in the contralateral VLPn, our results support the “LH inhibition” model. Hence, we conclude that the contralateral suppression occurs at the presynaptic site of VLPn, namely at the level of the PN-to-VLPn synapse within the LH. A similar mechanism of information sharing between the two brain hemispheres has recently been reported for the mouse olfactory system, where particular neurons interconnect mirror-symmetric mitral/tufted cells of the two olfactory bulbs (Grobman et al., 2018).

## Conclusion and Future Perspective

In this perspective article, we aimed to investigate the neuronal response of higher-order neurons, VLPn, to a lateralized olfactory stimulus. We demonstrate that activation of these neurons induces contralateral inhibition. This inhibition occurs most likely presynaptically to the VLPn in the LH. In addition, this contralateral suppression may contribute to flies’ navigation behavior following the concentration gradient across the two antennae. However, navigation toward (or away from) an odor involves the integration of multimodal sensory information (Baker and Hansson, 2016). Chemotaxis uses, besides olfactory information, visual and mechanosensory cues. Interestingly, the VLP neuropil receives inputs from neurons of all three sensory modalities (Lai et al., 2012; Zhu, 2013; Zhou et al., 2015), and thus represents a putative brain region for multimodal integration. For a future perspective, it would be highly interesting to investigate the role of VLPn for integrating visual and mechanosensory information along with the olfactory input. Moreover, it will be intriguing to manipulate the activity of VLPn of only one side of the brain, using a genetic approach (Wu et al., 2016), and to observe the behavioral consequences regarding navigation along odor plumes.

## Materials and Methods

### Flies Stocks

Flies were reared on standard cornmeal molasses medium under 12 h/12 h light/dark cycle at 25°C. Four to six days old adult females were used for calcium imaging experiments, and 5–10 days old flies were used for the immunostaining. The following stocks were used: *NP5194-Gal4* (gift from Greg Jefferis), *20XUAS-IVS-GCaMP6f* (VK00005) [Bloomington Drosophila Stock Center (BDSC) 52869], and *UAS-mCD8-GFP* (BDSC 5137).

### Immunostaining and Confocal Microscopy

Immunostaining was performed as previously described (Vosshall et al., 2000). In brief, brains were dissected in phosphate-buffered saline (PBS) (Ca^2+^, Mg^2+^ free) in room temperature, and then fixed in 4% paraformaldehyde (PFA) in PBS for 30 min at 25°C. Afterward, the brains were washed three to four times for 1.5–2 h in total in PBS-T (PBS + 0.3% Triton X-100) and blocked for 1 h in PBS-T + 4% normal goat serum (NGS) at room temperature. Brains were then transferred into primary antibody diluted in PBS-T + 4% NGS and incubated for 48 h at 4°C. Then, brains were washed three to four times in PBS-T at 25°C before incubation in secondary antibody for 24 h at 4°C. After secondary antibodies, brains were mounted in VectaShield (Vector Labs) after three to four times for washing with PBS-T. Stained brains were acquired with Zeiss LSM 880 with a 40× water immersion objective lens. The following primary antibodies were applied: chicken anti-GFP (1:500, Life Technologies) and mouse anti-nc82 [1:30; Developmental Studies Hybridoma Bank (DSHB)]; secondary antibodies are Alexa Fluor 488 goat anti-chicken (1:300, Life Technologies) and Alexa Fluor 633 goat anti-mouse (1:300, Life Technologies).

### Two-Photon Calcium Imaging

*In vivo* preparation of the flies for calcium imaging was previously described in Strutz et al. (2012). In short, flies were briefly anesthetized on ice and fixed with the neck onto a custom-made Plexiglas mounting block with copper plate (Athene Grids, Plano) and a needle before the head to stabilize the proboscis. Head was glued to the stage using Protemp II (3 M ESPE). We added Ringer’s solution [NaCl: 130 mM, KCl: 5 mM, MgCl_2_: 2 mM, CaCl_2_: 2 mM, sucrose: 36 mM, and HEPES–NaOH (pH 7.3): 5 mM] (Estes et al., 1996) to the head. A small window was cut in the fly’s head to expose the underneath brain. Care was taken while removing all fat, trachea, and air sacs to reduce light scattering.

Ventrolateral protocerebrum neurons were imaged using two-photon laser scanning microscope (2PCLSM, Zeiss LSM 710 meta NLO) equipped with an infrared Chameleon Ultra^TM^ diode-pumped laser (Coherent, Santa Clara, CA, United States) and a 40× water immersion objective lens (W Plan-Apochromat 40×/1.0 DIC M27). The microscope and the laser were placed on a smart table UT2 (New Corporation, Irvine, CA, United States). The fluorophore of GCaMP was excited with 925 nm. Fluorescence was collected with an internal GaAsP detector. For each individual measurement, a series of 40 frames acquired at a resolution of 256 × 256 pixels was taken with a frequency of 4 Hz. Odors were applied during frames 8–14 (i.e., after 2 s from the start of recording for 2 s); 1.5–2 min of clean air was applied between odors, in order to flush any residues of odors and to let the neurons go back to its resting phase. Odor source was lateralized by removing one antenna just before imaging. The identity of the intact antenna was pseudo-randomized between preparations.

### Odor Delivery System

Pure compounds were diluted in mineral oil and were freshly prepared after approximately 1 week. Fifty milliliters of glass bottles with custom-made lid insert (POM; HL Kunststofftechnik, Landsberg, Germany) were equipped with push-in adapter (jenpneumatik & Schlauchtechnik GmbH, Jena, Germany) for the tubing system. Odors were delivered via Teflon-tubes (jenpneumatik & Schlauchtechnik GmbH, Jena, Germany) and were changed for each odor to avoid contamination. For controlling the odor delivery, we used the LabVIEW software (National Instruments) which was connected to the ZEN software (Zeiss) to trigger both image acquisition as well as odor delivery. A continuous airstream, whose flow of 1 L min^–1^ was monitored by a flowmeter. A peek tube guided the airflow to the fly’s antennae. Behind the chamber with the fly was an air extraction system (flow rate 5 L min^–1^) to prevent contamination of the room air.

### Functional Imaging Data Analysis

Functional imaging data were analyzed using ImageJ^1^. All recordings were corrected for movement using a plugin in ImageJ. A region of interest was assigned on the LH of each animal and the change in fluorescence was measured. The raw fluorescence signals were converted to Δ*F*/*F*_0_, where *F*_0_ is the averaged baseline fluorescence values of 2 s before the odor onset (i.e., 0–7 frames). For the average Δ*F*/*F*_0_, average of frames 11–18 was calculated for each trail and averaged among trails. The VLPn could be reliably identified from the fluorescence baseline of GCaMP6f.

### Laser Transection

Transections of the VLPn tract were performed in one brain hemisphere. Using the baseline fluorescence of GCaMP at 925 nm, we selected a transection area (∼30 μm in a single focal plane) on the tract few micrometers before its entry site into the LH. The transection area was continuously illuminated with 760 nm for 1 min. To confirm a complete lesion of the VLPn tract, a fast z-stack with 925 nm was generated. Successful transection resulted in a small cavitation bubble (Vogel and Venugopalan, 2003). After transection was complete, we left the animal for 5 min for neuronal recovery before continuing with calcium imaging.

## Data Availability

All datasets generated for this study are included in the manuscript and/or the supplementary files.

## Author Contributions

AAMM and SS conceived and designed the study. AAMM performed all the experiments. AAMM and SS analyzed the data and interpreted the results. BSH provided intellectual and financial support. AAMM, BSH, and SS wrote the manuscript.

## Conflict of Interest Statement

The authors declare that the research was conducted in the absence of any commercial or financial relationships that could be construed as a potential conflict of interest.

## Footnotes

1 https://fiji.sc/
